# The Evolution of Multivariate Maternal Effects

**DOI:** 10.1371/journal.pcbi.1003550

**Published:** 2014-04-10

**Authors:** Bram Kuijper, Rufus A. Johnstone, Stuart Townley

**Affiliations:** 1 Environment and Sustainability Institute, University of Exeter, Penryn, United Kingdom; 2 Behaviour and Evolution Group, Department of Zoology, University of Cambridge, Cambridge, United Kingdom; 3 CoMPLEX, Centre for Mathematics and Physics in the Life Sciences and Experimental Biology, University College London, London, United Kingdom; 4 Department of Genetics, Evolution and Environment, University College London, London, United Kingdom; Pennsylvania State University, United States of America

## Abstract

There is a growing interest in predicting the social and ecological contexts that favor the evolution of maternal effects. Most predictions focus, however, on maternal effects that affect only a single character, whereas the evolution of maternal effects is poorly understood in the presence of suites of interacting traits. To overcome this, we simulate the evolution of multivariate maternal effects (captured by the matrix **M**) in a fluctuating environment. We find that the rate of environmental fluctuations has a substantial effect on the properties of **M**: in slowly changing environments, offspring are selected to have a multivariate phenotype roughly similar to the maternal phenotype, so that **M** is characterized by positive dominant eigenvalues; by contrast, rapidly changing environments favor **M**s with dominant eigenvalues that are negative, as offspring favor a phenotype which substantially differs from the maternal phenotype. Moreover, when fluctuating selection on one maternal character is temporally delayed relative to selection on other traits, we find a striking pattern of cross-trait maternal effects in which maternal characters influence not only the same character in offspring, but also other offspring characters. Additionally, when selection on one character contains more stochastic noise relative to selection on other traits, large cross-trait maternal effects evolve from those maternal traits that experience the smallest amounts of noise. The presence of these cross-trait maternal effects shows that individual maternal effects cannot be studied in isolation, and that their study in a multivariate context may provide important insights about the nature of past selection. Our results call for more studies that measure multivariate maternal effects in wild populations.

## Author Summary

In numerous organisms, mothers influence the phenotype of their offspring by transmitting hormones, antibodies and nutrients to the embryo. Evolutionary studies that make predictions about the evolution of these maternal effects typically focus, however, on single maternal characters only, in isolation of other traits. This contrasts with insights from quantitative genetics where reliable predictions about evolutionary change can only be made when measuring multiple traits simultaneously. The current study is therefore the first to make formal predictions about the evolutionary properties of multiple maternal effects. We show that maternal phenotypic characters generally give rise to developmental interactions in which one maternal character affects multiple offspring characters. In turn, such interactions can give rise to correlations between different traits in parent and offspring, which constrain evolutionary responses to sudden change. In addition, we find that the rate of environmental change directly affects some of the measurable properties of maternal effects: in rapidly changing environments, multivariate maternal effects are negative, so that offspring attain phenotypes that are different from their mothers, whereas positive maternal effects where offspring are more similar to their mothers occur in slowly changing environments. Hence, multivariate maternal effects provide a clear signature of the past selective environment experienced by organisms.

## Introduction

Since selection often varies both over space and time [1]–[3], evolutionary mechanisms that increase adaptation to changing environments are considered to be highly advantageous [4], [5]. Conventional studies focus on mechanisms such as bet-hedging [6]–[8] and, in particular, within-generational phenotypic plasticity [5], [9]–[11] as major adaptations to changing environments. However, a growing number of recent studies suggest that nongenetic effects provide an additional way of adaptation to changing environments [12]–[14]. Here, nongenetic effects refer to any effect on the offspring phenotype that is brought about by the transmission of factors (other than sequences of DNA) from parents or more remote ancestors to the offspring [13], [15]. Nongenetic effects can be realized through a variety of mechanisms, such as social learning [16], the transmission of DNA methylation variants [17] or the transmission of maternal factors such as antibodies or hormones [18], [19]. Importantly, when nongenetic effects are present in a population, an individual's phenotype becomes a function of the phenotypes (or the environment) of its parents or previous ancestors, giving rise to a form of transgenerational plasticity that is suggested to allow for increased flexibility when coping with environmental change [20]–[22].

Theoretical studies indeed predict that nongenetic effects are selectively favored in fluctuating environments [23]–[25], particularly when the parental phenotype provides information about selective conditions encountered by future generations [23], [24]. The role of parental information has been particularly well studied in the context of maternal effects, whereby the maternal phenotype or environment affects the offspring's phenotype through the provisioning of resources, antibodies or hormones [26]–[29]. However, most studies on maternal effects focus mainly on univariate scenarios, in which a single maternal factor influences a single offspring character. By contrast, studies in plants and animals suggest that maternal effects typically have a multivariate nature, involving suites of interacting parental and offspring characters (e.g., [30]–[33]). Indeed, this multivariate nature has long been appreciated by those models that assess the consequences of (non-evolving) nongenetic effects to phenotypic evolution [34]–[38]. As yet, however, no theoretical predictions exist about the evolution of these multivariate maternal effects themselves.

We believe that taking a multivariate view on the evolution of maternal effects is insightful for at least two different reasons. First, as stated before, the main prediction of univariate models is that maternal effects evolve when the parental phenotype correlates with selective conditions encountered by offspring [23], [24], [29], [39]. It is currently unclear how these predictions play out when offspring are not influenced by a single, but multiple components of a parental phenotype, raising the question how offspring should weigh information that results from the presence of multiple maternal effects (e.g., the presence of multiple maternal hormones and immonoglobulins in avian eggs [33], [40], [41]). A second reason for considering the evolution of nongenetic effects in a multivariate context is the finding [42] that the multivariate configuration of all maternal effects (here assumed to be captured in the matrix **M**, [34], [36], [43]) plays an analogous role in determining the course of evolution as the genetic variance-covariance matrix **G** that describes the scope for correlated selection between traits [44]–[46]. The role of the **G**-matrix in multivariate evolution has been the focus of a vast body of research for the last 40 years, and a substantial set of predictions exists on the ecological and social contexts that give rise to specific configurations of **G** (e.g., [47]–[49]) and ensuing evolutionary constraints [46], [50]–[54]. By contrast, there are yet no studies which investigate the selective conditions that lead to different configurations of **M**, which may similarly constrain phenotypic evolution [25], [34], [42]. As a first step towards a more inclusive theory that aims to incorporate both nongenetic and genetic constraints on evolution, the current study therefore aims to investigate which structures of **M** are likely to evolve across different ecological contexts.

To this end, we develop a formal model to make predictions about the evolution of multiple maternal effects in a periodically fluctuating environment. In the current model, a maternal effect reflects any causal influences of the maternal phenotype on the offspring's phenotype [18], [55]. Prominent examples of maternal effects are the modulation of offspring phenotypes by the maternal adjustment of a variety of egg hormones [41], [56] or the transmission of maternal antibodies to the embryo [57], [58]. In the current study, we focus on the evolution of multivariate maternal effects in the context of fluctuating selection. Specifically, we are interested in scenarios where the nature of selective fluctuations diverges between different maternal characters. For example, different maternal traits endure selection at different timepoints, because some maternal traits endure selection at different seasons than others, or because some selection on some characters may be more predictable (i.e., less stochastic noise) than selection on other characters. The current study thereby investigates whether such contexts lead to the interaction among different maternal characters, which cannot be inferred from studying the evolution of single traits in isolation.

We study the evolution of multivariate maternal effects in a fluctuating environment using individual-based evolutionary simulations. Although analytical approaches would be preferred to provide a general evolutionary model of multivariate phenotypes, an analytical assessment of multivariate evolution quickly becomes prohibitively difficult, even when only the evolution of the genetic variance-covariance matrix **G** is considered (while maternal effects are absent). It is thus no surprise that individual-based simulations have been the method of choice when considering more complicated scenarios of multivariate evolution (e.g., [49], [59]–[61]). Moreover, maternal effects involve the additional complexity that phenotypic evolution depends on past generations, so that an analytical description requires a number of strong equilibrium assumptions (such as a constant covariance between genotypes and phenotypes, [34], [43]). Here, however, we use the flexibility of individual-based, evolutionary simulations to study the evolution of maternal effects in a dynamical fashion without these limiting assumptions.

## Models

**Figure 1:**
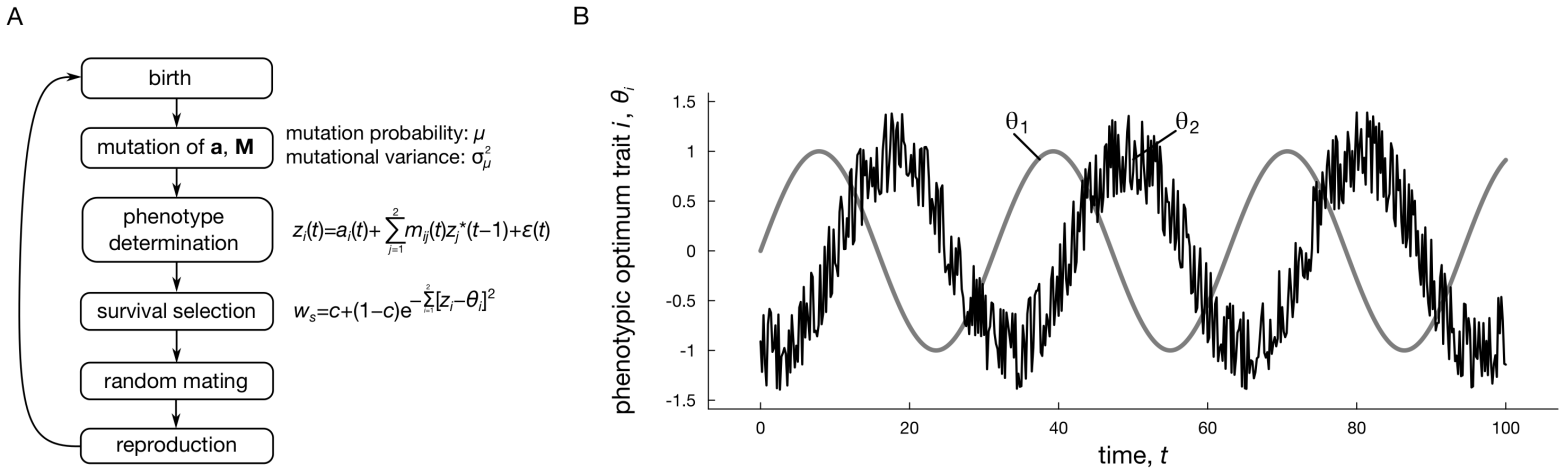
Graphical description of the model (panel A) and an example scenario in which fluctuating selective optima *θ*_1_(*t*) and *θ*_2_(*t*) fluctuate over time (panel B). In this particular scenario, optimum 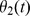 is delayed relative to 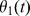 with phase shift *φ* and 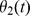 contains noise (indicated by noise parameter *d*). The amount of noise in 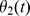 is proportional to the disturbance parameter *d*. Parameters: 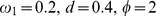.

Our model assumes a sexually reproducing population of *N* = 5000 randomly mating hermaphrodites having discrete, nonoverlapping generations. A graphical summary of the life cycle is given in Figure 1A.

### Gene loci

To assess the evolution of multiple maternal effects, we study the simplest possible case, in which an individual that breeds in generation *t* expresses two phenotypic traits, 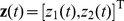 (where T denotes transposition). Each individual bears six unlinked, diploid loci: two of which correspond to the breeding values of both phenotypic characters 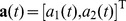, that determine the baseline elevation of both phenotypic traits. The remaining four loci code for the entries of the 2× maternal effect matrix 
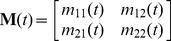
, of which the entry 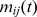 reflects the maternal influence from maternal phenotype *j* to offspring phenotype *i* (see eq. [1] below). For all six loci, the two alleles at each locus interact additively (i.e., dominance effects are absent) and inheritance is biparental. During each generation, each allele mutates with probability 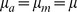, upon which a value drawn from a normal distribution with mean 0 and variance 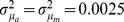 is added to the current allelic value (i.e., a continuum of alleles model [62]). Each individual simulation run started from the initial values 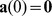 and 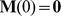 continued for 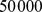 generations. Throughout, we found that values of 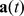 and 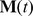 attained stable values after approximately a tenth of this total timespan (e.g., see Figure S4).

### Phenotypes

Extending seminal quantitative genetics models that focus on non-evolving multivariate maternal effects [34], [35], [43], [63]–[65], the phenotype 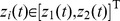 of an individual in generation *t* is then given by the following recursion:
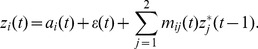
(1)Here, 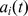 is the breeding value of trait 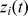, while *ε*(*t*) reflects the contribution of environmental noise, which is normally distributed with mean 0 and variance 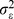 as in classical quantitative genetics models [44], [66]. The starred value 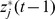 denotes the value of the maternal phenotype 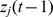 after selection. As noted before, 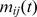 is the evolving maternal effect coefficient, describing how maternal character 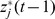 influences offspring phenotype *i*. Note that we assume that the maternal effect loci are controlled by the offspring, representing a scenario in which entry 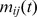 of **M**(*t*) reflects the offspring's sensitivity to maternal trait *z_j_* [67], [68]. For example, the vector **z**(*t*) may reflect titers of different offspring hormones, and the entry 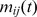 specifies how the maternal titer 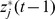 of hormone *j* (e.g., measured during pregnancy or added to the egg) affects the titer 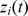 of offspring hormone *i* (e.g., mediated by the number of hormone binding sites present in the offspring endocrine cells). Indeed, studies indicate that maternal hormone and protein titers affect offspring hormone concentrations, often in a multivariate fashion (e.g., [33], [56], [69]).

While multivariate maternal effects **M**(*t*) are assumed to be genetically expressed by the offspring, they still give rise to nongenetic effects on the multivariate phenotype **z**(*t*). This is because the offspring's phenotype is not merely influenced by **M**(*t*) itself, but the product 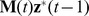 of maternal effects and the parental phenotype. Consequently, the involvement of the maternal phenotype gives rise to the well-known ‘cascading nature’ of maternal effects [34], [35], [70], whereby the maternal phenotype 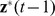 is itself a function of the multivariate grandmaternal phenotype 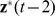, which in turn is a function of the phenotypes 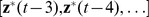 of previous ancestors. As the offspring's phenotype **z**(*t*) is thus not only a function of DNA sequences it received from its ancestors (i.e., genes coding for **a**(*t*) and **M**(*t*)), but also of the phenotypes of its ancestors, **M**(*t*) gives rise to nongenetic effects on the offspring phenotype (see also [38]).

### Fluctuating survival selection

After birth and phenotype determination (as in eq. 1), each newborn enters a survival stage during which it endures survival selection. The survival probability *w_s_* decreases nonlinearly with a displacement of the character 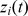 away from the selective optimum 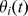, according to the Gaussian function
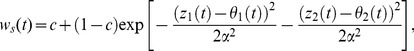
(2)where all individuals are assumed to have a baseline survival probability of *c*, while the strength of stabilizing selection on both phenotypes is proportional to the remainder 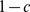. *α* ^2^ measures the width of the selection function and is therefore inversely proportional to the strength of selection. Throughout, we assume that 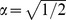.

#### Periodic fluctuations

**Figure 2:**
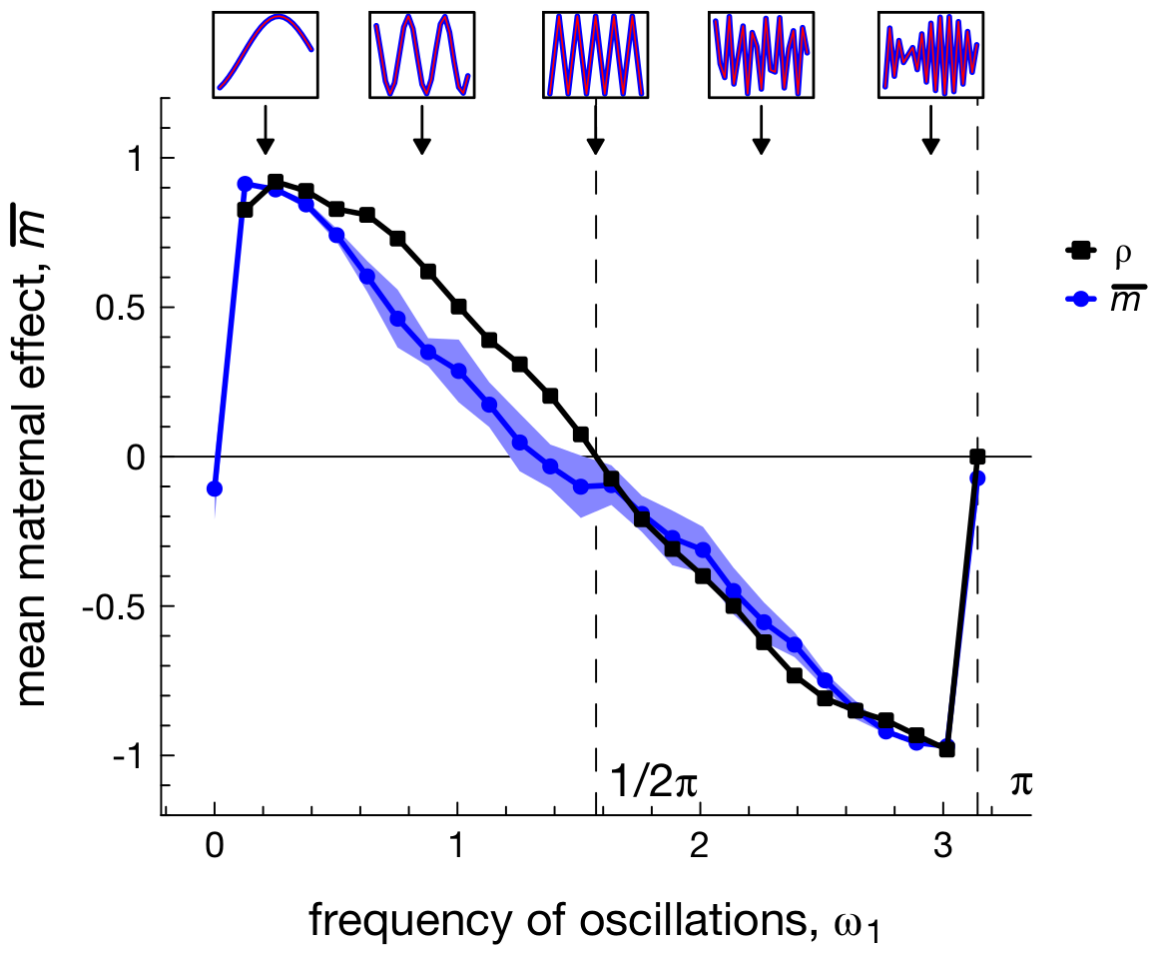
The evolution of univariate maternal effects for different frequencies *ω*_1_ of periodically fluctuating selection. Each dot represents the average maternal effect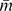 measured over ten replicate simulations (at generation 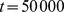), while the shaded areas depict corresponding standard deviations. Boxes on the top of the graph depict the a snapshot in time of the fluctuations in the selective optimum *θ*(*t*) over the course of 20 generations. Note that any fluctuations are absent whenever *ω*_1_ is a multiple of *π*, as the environment then fluctuates back towards the same state (i.e., Δ*θ* = 0) before selection takes place in the next generation. Black lines depict the autocorrelation 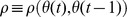 of selective conditions between the parental and offspring generations. Parameters: 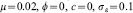.

Throughout the main part of the paper, optima 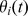 are assumed to fluctuate according to a discrete-time sinusoid
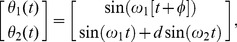
(3)where the optimum *θ_i_* of trait *z_i_* fluctuates periodically with amplitude *ω*_1_, mimicking, for example, a seasonal environment or periodic environmental forcing on longer timescales (e.g., ‘El Niño,’ [71]). The rate at which the selective environment fluctuates is given by *ω*_1_. In addition, both fluctuating optima can either be *φ* time steps delayed relative to each other, or fluctuations in one optimum can be characterized by a certain degree of disturbance (*d*>0), through the presence of additional (stochastic) fluctuations with a frequency of *ω*_2_, where values of *ω*_2_ are chosen from a uniform distribution between 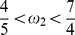 (see Figure 1 for an example). Note that discrete-time sinusoids give rise to more complicated periodic behaviors in comparison to their continuous-time counterparts, since they typically lack strict periodicity. Consequently, timeseries of selective optima generally look more erratic and variable over short-term periods (e.g., see insets in Figure 2), which is likely to more closely correspond to temporal variation observed in natural timeseries.

Finally, because selective optima fluctuate around 0 (i.e., no directional selection), we found no significant evolutionary change in the breeding values 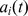 underlying each phenotype 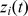 [72] (e.g., see Supplementary Figure S4A). We therefore only focus on the evolved values of 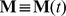 in the main part of this study.

#### Stochastic fluctuations

In addition to the periodic fluctuations, the Supplementary Figures consider a scenario in which *θ_i_* fluctuates according to a stochastic process rather than a sinusoid function. Simulations are identical to the periodic case, except that *θ_i_* is now drawn from a Gaussian autocorrelated time series with mean 0 and variance 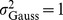, whilst varying the value of the autocorrelation 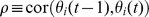 or cross-correlation 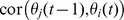 in selective conditions between two subsequent generations (see Results and Supplementary Figures S1, S2B, S3 and S5).

### Reproduction

After survival selection, the 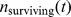 surviving individuals reproduce: each surviving individual produces a number of *n* = 10 ova, which are all fertilized by a randomly chosen other survivor that acts as a sperm donor. From the zygotes, new individuals are sampled with replacement to maintain a population of constant size *N*. Subsequently, the cycle starts anew with mutation and phenotype determination, as depicted in Figure 1A.

## Results

### Result 1: The shape of the maternal effects matrix M is determined by the rate of environmental change

#### The evolution of a single maternal effect, *m*

To understand the evolution of multiple maternal effects in a fluctuating environment, it proves illustrative to first investigate the evolution of a single phenotypic trait *z* that is influenced by single a maternal effect, *m* (Figure 2). When selective conditions fluctuate at a slow rate (small rate of change, *ω*_1_), selection between two consecutive generations is likely to be positively correlated (autocorrelation 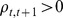, black line in Figure 2). Consequently, offspring are selected to develop a phenotype of a similar sign as their mother, leading to the evolution of positive values of the maternal effect *m*. Faster fluctuations, however, (i.e., 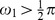) lead to negative autocorrelations (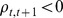) in selective conditions between mother and offspring. As a result, offspring are selected to have phenotypes of a different sign in comparison to the maternal phenotype, selecting for negative maternal effects (*m*<0, Figure 2).

When environmental change is effectively absent (i.e., when the frequency of change is *ω*_1_ = 0 or when exactly half a period of fluctuations has passed before selection takes place in the next generation: 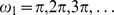), we find that *m* evolves to negative values. The evolution of *m*<0 in the absence of environmental fluctuations is in line with previous findings [25], [73], which predict that slightly negative values of *m* are optimal in stable environments, as a means to reduce the amount of phenotypic variance. It also explains why *m* has a smaller absolute magnitude when it evolves to positive values in comparison to cases where *m* evolves to negative values (see Figure 2), as positive *m* are more likely to increase, rather than decrease the phenotypic variance around the selective optimum [25], [34].

So far, we have only studied a periodic environment, which raises the question whether these conclusions can be generalized to stochastic environments. In Supplementary Figure S1, we study the evolution of *m* when fluctuations in the selective optimum *θ*(*t*) are given by a Gaussian time series. Because the frequency parameter *ω*_1_ does not apply to stochastic environments, here we therefore vary the autocorrelation *ρ* between two subsequent time steps directly. Figure S1 shows that positive values of *m* occur when fluctuations are characterized by positive autocorrelations *ρ*, while negative values of *m* evolve when *ρ*<0. Hence, a marked correspondence exists between the degree of environmental autocorrelation and the sign and magnitude of *m* in both periodic and stochastic environments (see Figure 2).

#### The evolution of multiple maternal effects, M

When extending our model to allow for multiple, coevolving maternal effects, we first focus on the simplest possible case in which fluctuations in selective conditions are identical for both phenotypic characters *z*_1_ and *z*_2_. When only those maternal effects that affect the same trait in offspring (i.e., 
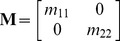
) are allowed to evolve, we find an identical outcome as in the previously discussed case with a single maternal effect (see Figure S2A): both entries *m*_11_ and *m*_22_ evolve to positive values in slowly changing environments (that are characterized by positive autocorrelations), and to negative values in rapidly changing environments (characterized by negative autocorrelations). Figure S2B shows that also in a stochastic environment, negative and positive maternal effects evolve dependent on the value of the autocorrelation between selective optima in parents and offspring.

**Figure 3:**
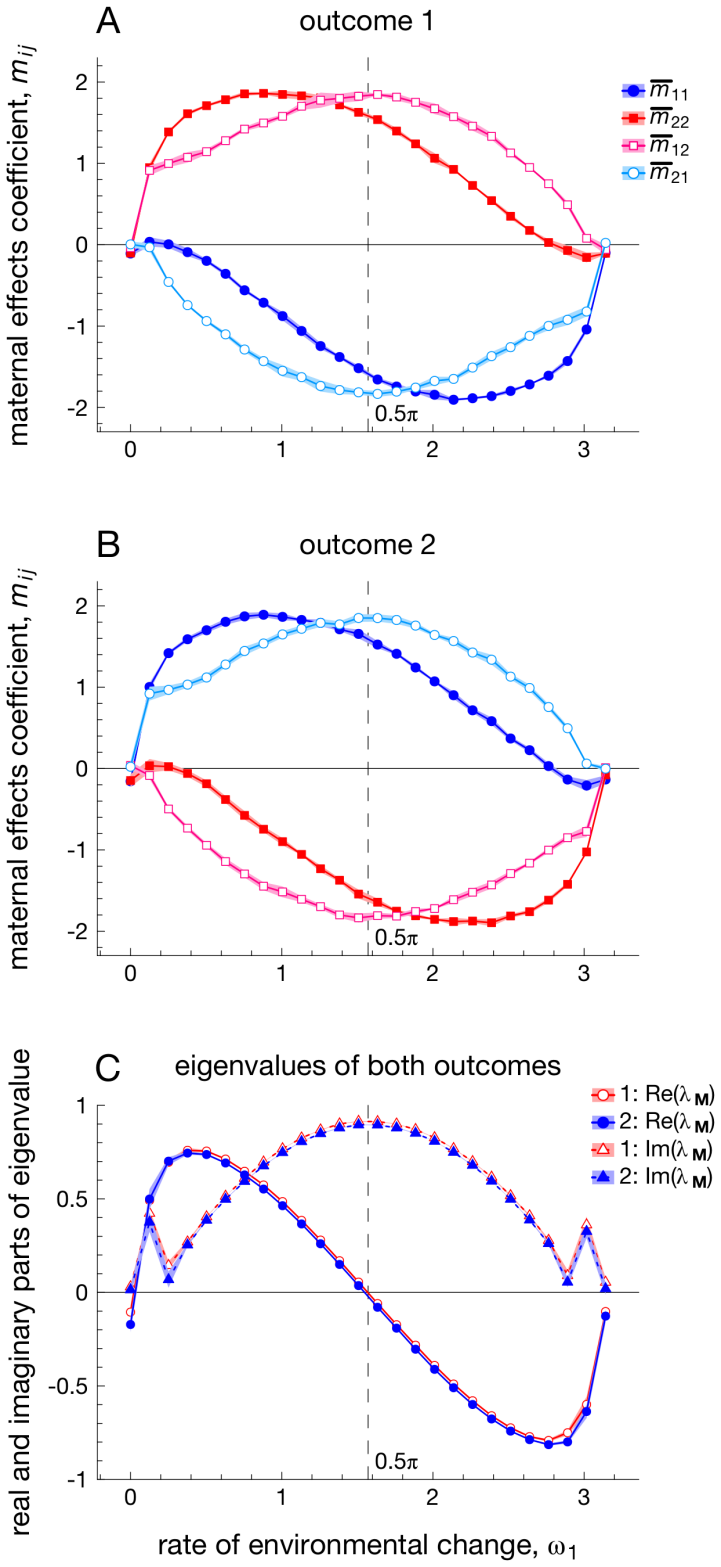
The evolution of multivariate maternal effects for different frequencies *ω*_1_ of environmental change, when periodically fluctuating selection is identical across traits (i.e., *θ*_1_(*t*) = *θ*_2_(*t*)). Panels A, B: entries of the multivariate maternal effects matrix **M** evolve towards two alternatively stable outcomes (outcome 1 and outcome 2). Panel C: real and imaginary parts of the eigenvalue 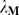 of the maternal effects matrix are identical for both outcomes. For the sake of clarity, a value of 0.01 was subtracted from the eigenvalues of outcome 2, to prevent overlap with the eigenvalues in outcome 1. Each point in panels A and B depicts values of the maternal effect 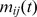 (at generation 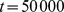) averaged over ten replicate generations, while shading depicts corresponding standard deviations. Parameters: 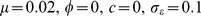.

When all entries of 
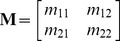
 are able to evolve, outcomes differ from the univariate case depicted in Figure 2. Figures 3A and 3B show that all maternal effects either attain positive or negative values (of a substantial magnitude) for a broad range of rates of change *ω*_1_. Moreover, Figure 3 shows that entries of **M** evolve to two alternatively stable, evolutionary outcomes: despite identical starting conditions, half of all replicate simulations result in *m*_11_ and *m*_21_ having negative values (while *m*_22_ and *m*_12_ are positive, Figure 3A), whereas the other replicates result in *m*_11_ and *m*_21_ having positive values (with *m*_22_ and *m*_12_ being negative, Figure 3B).

Both these alternatively stable outcomes result, however, in a similar overall ‘shape’ of the maternal effects matrix **M**, when measured in terms of its dominant eigenvalue 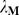: Figure 3C shows that real (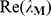) and imaginary (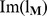) parts of the eigenvalue are the same for alternative evolutionary outcomes. Indeed, it is this shape of **M** that determines how the maternal phenotype influences offspring phenotypes. For example, a positive real part of the eigenvalue implies that offspring will have a multivariate phenotype 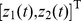 of a similar sign as the maternal phenotype, which is advantageous when selective optima are unlikely to change much between generations (left part of Figure 3C). By contrast, negative values of 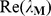 imply that offspring favor phenotypes that are different in sign to the maternal phenotype, which is beneficial when the selective environment changes rapidly between consecutive generations (right part of Figure 3C). Since the real part of 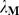 is given by 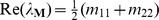, interchanging *m*_11_ and *m*_22_ gives rise to identical values, hence explaining why the two alternatively stable states lead to similar consequences.

Whereas the value of 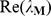 informs whether maternal effects give rise to switches in sign between offspring and maternal phenotypes, the imaginary parts 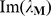 indicate changes in phenotypic values that span a larger number of generations [74], [75]. Although a full assessment of the dynamical consequences of 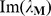 is beyond the scope of the current paper, we highlight the role of 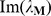 when it attains its largest magnitude, at the point 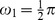 (Figure 3C). At this point, the autocorrelation between selective conditions experienced by mother and offspring is nearly absent 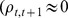), while the autocorrelation 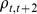 between selective conditions experienced by grandmothers and offspring is, in fact, at a minimum: 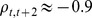. In other words, offspring would benefit substantially from a phenotype that is opposite in sign to that of their grandparents. Using results from standard calculus (e.g., p. 355 in [75]), it can be shown that the real and imaginary parts in Figure 3C at the point 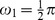 give rise to fluctuations in phenotypic values with a full period of 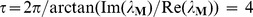 timesteps. A period of 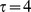 implies that it takes 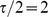 timesteps for a phenotype to change sign, which matches the change in environmental conditions between grandparental and offspring generations. Supplementary Figure S4 confirms this finding, by highlighting that the presence of all four entries of **M** leads to a substantial increase in mean fitness relative to scenarios in which only both diagonal entries of **M** are evolving (and where 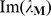 is necessarily 0).

Alternatively stable outcomes are also found when selective fluctuations are stochastic rather than periodic (Supplementary Figure S3), at least when selective optima are identical for both traits (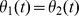, as previously considered for the periodic environment) (see Figures S3A–B). However, the eigenvalues of **M** (in particular their imaginary parts) are more modest in comparison to Figure 2C, as longer term fluctuations in a stochastic environment are inherently less predictable in comparison to a deterministic environment.

### Result 2: Temporally advanced selective conditions result in positive cross-trait maternal effects

**Figure 4:**
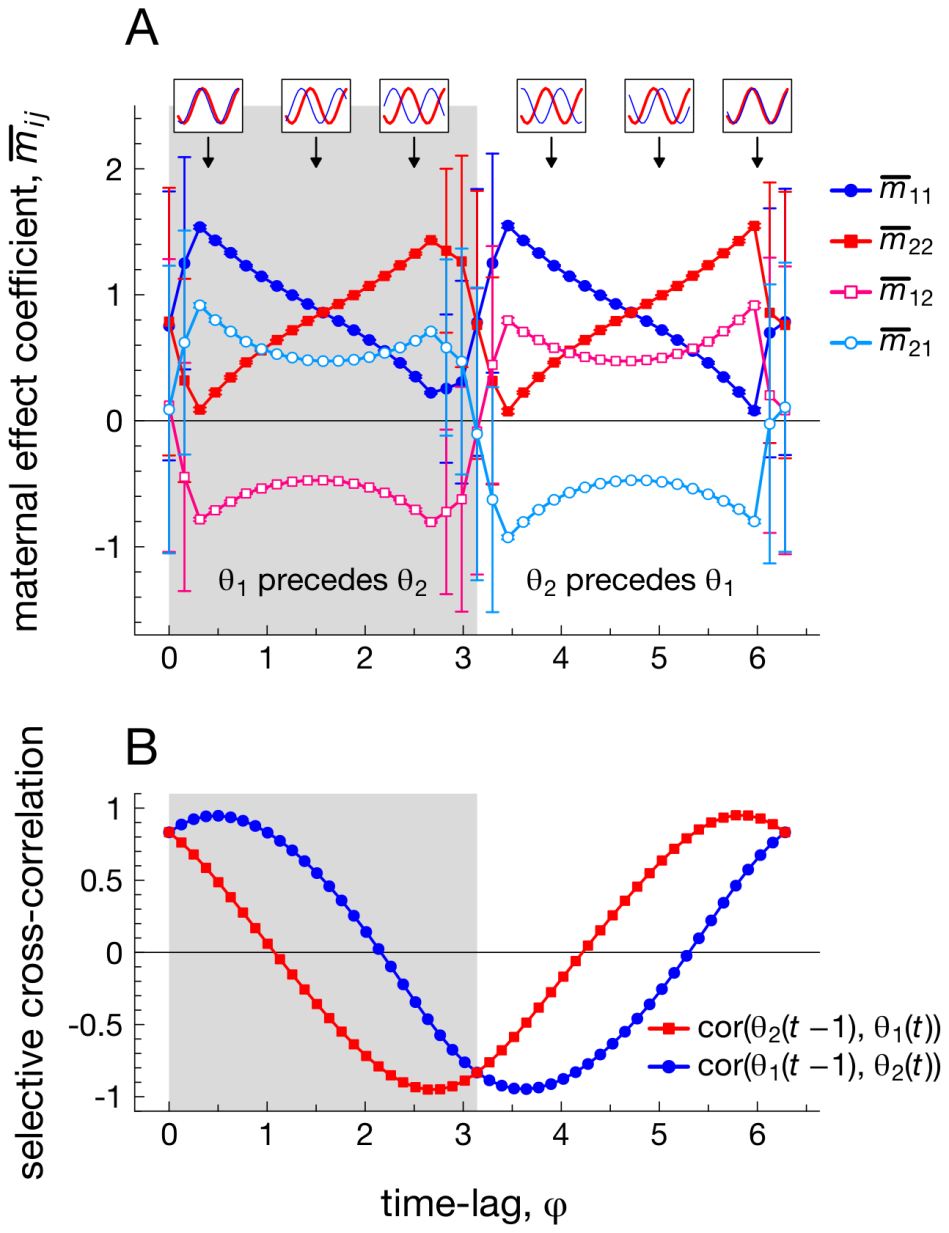
The evolution of multivariate maternal effects (panel A) when the evolutionary optimum *θ*_2_(*t*) lags *φ* timesteps behind the evolutionary optimum *θ*_1_(*t*). Grey area: fluctuations in optimum 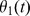 precede those of optimum 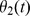 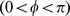. White area: 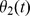 precedes 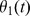 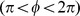. Insets on the top of the panel depict the fluctuating selective optima 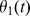 (blue line) and 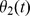 (red line) over time. Panel B: selective cross-correlations that result from varying the time-lag between both selective optima. Note that each point in panel A depicts the maternal effect 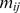 (at generation 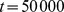) averaged over ten replicate generations, while error bars depict corresponding standard deviations. Error bars are large when time lags are small, as evolution then leads to alternatively stable states observed in Figures 3A,B. The results in this figure also extend to a periodic environment in which the time delay *φ* is stochastically distributed according to a normal distribution 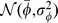 with mean 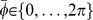 and variance 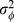 varying from 0.1 to 4 (results not shown). Parameters: 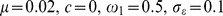.

So far, we have assumed that fluctuations in both selective optima 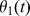 and 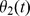 are identical, whereas different forms of fluctuating selection may act on each maternal trait, dependent on the ecological context that each trait experiences. Figure 4A shows, for example, how delays between the selective optima of both phenotypes impact on the evolution of multivariate maternal effects matrix **M**. First, the presence of a delay of *φ* timesteps between the two selective optima leads to a collapse from the two alternatively stable outcomes observed in Figure 3 to a single evolutionary outcome, unless both optima fluctuate in an exactly opposing fashion (depicted by the large error bars when 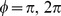). Second, Figure 4A shows that both cross-trait maternal effects (*m*_12_ or *m*_21_, which reflect maternal influences from one maternal trait to a different offspring character) evolve to opposing positive and negative values, dependent on the delay *φ* between both optima. The maternal effect *m*_21_ is positive (and *m*_12_ negative) when fluctuating selection on trait *z*_1_ is advanced relative to selection on *z*_2_ (grey region in Figure 4A), whereas *m*_21_ is negative (and *m*_12_ positive) when fluctuating selection on trait *z*_2_ is advanced relative to selection on *z*_1_.

Insights on the adaptive significance of cross-trait maternal effects can be derived from the cross-correlations 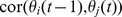 of selective conditions between different traits in parents and offspring, which reflect to what extent one maternal phenotype is able to predict future selection on a different offspring phenotype (Figure 4B). Focusing on the grey region in Figure 4B where the selective optimum 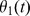 is advanced relative to 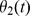, we find that 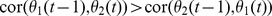. In other words, selection on maternal phenotype 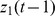 is overall positively associated to selection on offspring phenotype 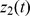 (or when it attains negative values these are always smaller in magnitude than the other cross-correlation). Since the evolving maternal effect *m*_21_ reflects the influence of maternal trait 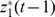 to offspring trait 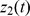, it evolves to positive values so that 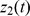 matches its future environment. By contrast, selection on the maternal phenotype 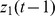 is overall negatively associated to selection on offspring phenotype 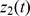 (or when it attains positive values these are always smaller in magnitude than the other cross-correlation). Hence, the maternal effect *m*_12_ evolves to negative values instead. The reverse scenario applies for the white region in Figure 3B, where selective optimum 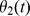 is advanced relative to 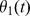. Indeed, we find that *m*_12_ then evolves to positive values, while *m*_21_ evolves to negative values. In other words, those maternal traits enduring fluctuating selection that is advanced relative to selection on other maternal traits are more likely to develop positive cross-trait maternal effects, whereas delayed fluctuating selection on maternal traits is more likely to lead to negative cross-trait maternal effects.

Lastly, we investigated whether such cross-trait maternal effects also evolve in multivariate stochastic environments. As cross-correlations are easily measurable in time series analyses [76], we chose to vary the cross-correlation directly in Figure S3 (although results are similar when adjusting the time-lag between both optima). In a stochastic environment without any cross-correlation between both selective optima, we find that cross-trait maternal effects *m_ij_* generally do not evolve, while the within-trait maternal effects *m_ii_* track the autocorrelation of its associated selective optimum *θ_i_* (Figure S3D,E). By contrast, when selective cross-correlations are nonzero, cross-trait maternal effects evolve to substantial values (Figure S5). For example, when varying the cross-correlation 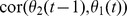 from −1 to +1, we find that the associated cross-trait maternal effect *m*_12_ evolves from strongly negative to strongly positive (Figure S5A). Instead, when varying the cross-correlation 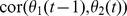, we find a similar pattern for the other cross-trait maternal effect *m*_21_. Similar to the periodic environment in Figure 4, we find that cross-trait maternal effects evolve in concordance to the prevailing cross-correlations between both fluctuating selective optima.

### Result 3: The strongest maternal effects evolve from maternal traits that endure little selective noise

Next, we assess the combined influence of environmental stochasticity and periodicity, by considering a scenario in which selection on trait *z*_1_ fluctuates only periodically whereas selection on trait *z*_2_ fluctuates both stochastically and periodically (e.g. see Figure 1). Such a scenario reflects, for example, a case in which some traits (e.g., *z*_1_) are associated with more predictable selective conditions than other traits (e.g., *z*_2_).

**Figure 5:**
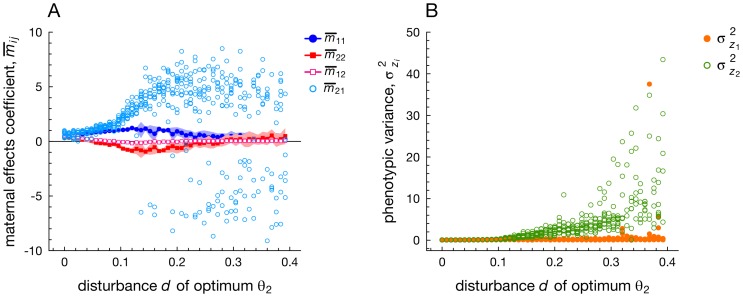
The evolution of multivariate maternal effects when varying the degree of noise in selective optimum *θ*_2_ while selective optimum *θ*_1_ remains noise-free. Panel A: evolving maternal effects. Panel B: corresponding phenotypic variances 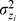 of each phenotypic trait, showing that phenotype *z*_2_ exhibits increased phenotypic variance (resulting in bet-hedging) when compared to *z*_1_. Parameters: 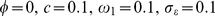.

In Figure 5A we vary the degree of stochastic noise *d* in selection acting on character *z*_2_. Increasing levels of noise lead to the evolution of substantial cross-trait maternal effects *m*_21_ from maternal character 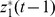 to offspring character 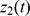. By contrast, the value of the other cross trait maternal effect *m*_12_ (from maternal character 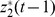 to offspring character 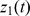) evolves towards zero. Both the same-trait maternal effects *m*_11_ and *m*_22_ evolve towards positive and negative values respectively, corresponding to a positive dominant eigenvalue 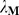 that is expected when the frequency of noise-free periodic fluctuations is relatively low (e.g., 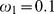 in Figure 5).

In order to explain the marked evolution of the cross-trait maternal effect *m*_21_, note that selection on maternal trait 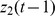 is characterized by white noise, while noise is absent on maternal trait 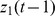. Consequently, maternal trait 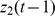 is a less reliable source of information about future selective conditions experienced by offspring. As a result, offspring are selected to obtain their information about periodic fluctuations from the other maternal trait 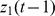, thereby evolving large, positive values of the cross trait maternal effect *m*_21_ while its counterpart *m*_12_ evolves towards a value of 0. Hence, asymmetries in information content between both maternal characters favor the evolution of cross-trait maternal effects from the most reliable maternal character, phenotype 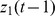.

When the degree of noise in *θ*_2_ increases to ever larger levels, we find that *m*_21_ attains either positive or negative values of a large absolute magnitude, 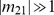 (Figure 5A). In line with analytical models that show that values of 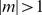 are associated with very large phenotypic variances [34], we find indeed that 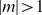 results in a large phenotypic variance of trait *z*_2_, 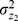, while the variance of the other trait 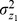 is generally small (Figure 5B). Large phenotypic variances are selectively favored in unpredictable environments, since a large diversity of phenotypes among siblings warrants the survival of at least a subset of them in the event of environmental change (bet-hedging: [6], [8], [77], [78]).

Given that maternal effects may thus drive the evolution of large phenotypic variances, large values of 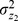 relative to 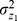 can only be realized by evolving a large cross-trait maternal effect *m*_21_. By contrast, evolving a large within-trait maternal effect *m*_22_ would also generate large values of 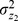, but also gives rise to highly detrimental carry-over effects in which extreme values of the maternal phenotype 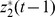 will lead to values of 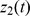 an even larger magnitude in the next generation. Such detrimental carry-over effects are absent for the cross-trait maternal effect *m*_21_, since it has the more constant maternal phenotype 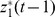 as its input. Hence, cross-trait maternal effects may facilitate the evolution of large phenotypic variances without giving rise to deleterious carry-over effects that are associated with large absolute values of within-trait maternal effects *m_ii_*.

## Discussion

Maternal effects give rise to an interaction between parental and offspring phenotypes that is considered to be an important adaptation to changing environments [19], [22], [25], [79]. Yet, few formal models predict how selection shapes the strength and sign of maternal effects in different ecological contexts (see [80] for similar remarks in the context of social interactions). The current study is therefore the first to show that different forms of environmental fluctuations can have a profound impact on the evolution of multivariate maternal effects.

When fluctuating selection acts in an identical fashion on all maternal traits, we find that the shape of the maternal effects matrix **M** (measured by its eigenvalues) evolves to be closely aligned with the degree of autocorrelation between parental and offspring phenotypes. Hence, we expect that slow fluctuations (leading to a positive autocorrelation) select for positive real parts of 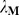, while rapid fluctuations select for negative real parts of 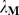. This finding corroborates a number of univariate models which showed that the rate of environmental fluctuations corresponds to the degree with which offspring should copy or diverge from their parent's phenotype in the case of discrete phenotypic variation [23], [39], [81]. Interestingly, our multivariate model shows that the same shape of **M** can sometimes be achieved in multiple ways, thereby leading to alternatively stable states in which entries of **M** can differ substantially in sign and magnitude between different subpopulations. Consequently, hybrid crosses between subpopulations are likely to lead to suboptimal values of maternal effects, potentially leading to postzygotic reproductive isolation. Nonetheless, as alternatively stable states collapse to a single outcome when fluctuating selection diverges between both traits, it remains to be seen whether maternal effects can indeed contribute to reproductive isolation across a broad range of contexts.

The most important result of the current study is that cross-correlations between selective fluctuations acting on different traits can select for striking configurations of cross-trait maternal effects. One way in which such cross-correlations can arise is due to time lags between selection acting on one trait and selection acting on another trait. Essentially, lag-times between fluctuating optima create asymmetries in the information content between both maternal characters, so that characters which endure selective fluctuations which are ‘advanced’ (relative to selective fluctuations on other traits) are more informative about future selective conditions than other characters. As a consequence, cross-trait maternal effects from such maternal traits should evolve to positive values, whereas cross-trait maternal effects based on traits enduring delayed selection should evolve to negative values. Hence, our study suggests that pairs of positive and negative maternal effects are indicative of lag-times (or cross-correlations in the action of fluctuating selection between different traits.

Another context in which asymmetries in information content occur between both maternal traits is when fluctuating selection on one character contains more stochastic noise relative to selection on other characters. In this case, we expect that offspring characters benefit from information contained in those maternal traits that endure the most predictable form of fluctuating selection, whereas offspring are selectively favored to ignore information from maternal characters with more selective noise when alternative maternal traits are available. Henceforth, we may expect cross-trait maternal effects to evolve that rely on those maternal traits which endure the most predictable forms of fluctuating selection. In addition, when stochastic noise in selective conditions acting on one particular trait is substantial, cross-trait maternal effects of large absolute magnitude may give rise to increased phenotypic variance in the trait that experiences noisy selection (see Figure 5B). Such increased phenotypic variation is selectively advantageous in fluctuating environments, as it gives rise to bet-hedging [6], [8], [82]. Following the seminal model of Kirkpatrick and Lande [34] our study indeed shows that large phenotypic variances are associated with relatively large magnitudes of maternal effects coefficients, i.e., 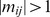. Henceforth, such ‘large’ maternal effects may provide an efficient means to increase phenotypic variation among offspring, which is in line with a number of studies that have identified maternal effects as a mechanism that gives rise to bet-hedging [23], [83], [84]. On the other hand, large maternal effects will also imply that any fluctuations in current trait values resonate to future generations, generating ever larger mismatches to future environmental conditions. To use maternal effects to increase phenotypic variance, individuals are selectively favored to use other maternal traits, which are less affected by stochastic fluctuations in selective conditions. Consequently, cross-trait maternal effects used in the context of bet-hedging are expected to rely on maternal characters that are stably inherited across generations.

While numerous empirical studies have measured nongenetic effects in animal and plant populations (reviewed in [15], [18], [79]), almost all of these studies have taken a univariate perspective and measured only single maternal effects coefficients [31], [85], [86], or alternatively, measured different maternal traits lumped into a single ‘maternal performance’ character ([30], [87]). Consequently, it is currently premature to assess whether our predictions correspond to any empirical measurements of maternal effects matrix **M**. The current study indicates, however, that overlooked components of **M**, such as cross-trait maternal effects, could potentially be an important adaptation to fluctuating selection, and may provide a signature of past selective differences between traits. We therefore hope that the current model provides an incentive for future studies to assess maternal effects in a multivariate context. Such measurements of **M** are facilitated by the recent advent of multivariate methods developed within the context of indirect genetic effects (IGEs, [87]–[90]) that allow for the estimation of **M** using variance components [91]. To our knowledge, only a single study so far has used these multivariate methods to measure **M**: Galloway and coworkers ([32], see also [70]) measured maternal effects across four different life history traits in the plant *Campanulastrum americanum*, finding that some cross-trait maternal effects have magnitudes similar to or sometimes even larger than any within-trait maternal effects on similar characters. Moreover, cross-trait maternal effects often differ considerably in magnitude and even in sign, suggesting that observed variability among components in **M** is present and might be matched to past selective conditions to test our hypotheses. Moreover, a follow-up study in the same population [70] highlights another distinctive feature of maternal effects, namely that evolutionary change in phenotypes in a given generation is the result of both current and past selection gradients. We therefore hope that more studies follow the example by Galloway, McGlothlin and coworkers [32] and measure maternal effects in multivariate contexts.

The current model has made a number of limiting assumptions that suggest possible directions for future work. The current study considers the simplest possible genetic architecture, where the breeding values 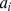 and maternal effects 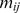 are represented by single, diploid loci. It is well established that the relative number of loci coding for each trait may affect the magnitudes of the additive genetic (co)variances (e.g., [92], [93]), which in turn may either enhance or constrain adaptation [46]. Apart from the potential effects of additive genetic (co)variances, however, we note that previous comparisons of multilocus and single-locus approaches (e.g., [94]–[97]) have shown that evolutionary endpoints are often remarkably similar regardless of the approach taken, so we expect the evolution of **M** to be robust to more complicated genetic architectures. Future studies should assess whether this prediction indeed bears out.

In addition, we exclusively focus on the evolution of cross-trait maternal effects due to temporal cross-correlations, thereby generating interactions between two different characters in a transgenerational context. By contrast, conventional studies on multivariate evolution focus exclusively on genetic correlations as the main (within-generational) signature of interactions between traits [98]–[102]. It would therefore be interesting to investigate whether those selective contexts that give rise to genetic correlations, such as trade-offs [103], [104], phenotypic plasticity [105], [106], developmental interactions [99], [100], [102] or sexual selection [97], [101], [107] also affect the evolution of cross-trait maternal effects.

In turn, the potential role of cross-trait maternal effects as constraints on phenotypic evolution is currently virtually unexplored [35], as conventional studies have focused almost exclusively on genetic correlations that constrain the multivariate response to selection [45], [46], [54]. When cross-trait maternal effects are present, however, past selection on a particular character in the previous generation may lead to a correlated selective response on other characters even when genetic correlations themselves are absent [34], [35]. In addition, a recent study on univariate maternal effects shows that maternal effects expose populations to stronger transient perturbations in response to sudden environmental shifts than populations without maternal effects [25], [73]. More work is therefore needed to single out those ecological conditions in which significant cross-trait maternal effects are expected to evolve, as well assessing their consequences to phenotypic adaptation.

## Supporting Information

Figure S1The evolution of a single maternal effect *m* when fluctuations in *θ*(*t*) are stochastic rather than periodic. Instead of varying the frequency of environmental change *ω*_1_ (which is only relevant to periodic environments), we now vary the autocorrelation *ρ* between selective conditions experienced by mother and offspring. Maternal effects evolve to be positive when *ρ* attains large, positive values. By contrast, negative values evolve for smaller values of *ρ*. Each dot represents the average maternal effect 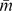 measured over ten replicate simulations (at generation 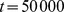), while the shaded areas depict corresponding standard deviations. Parameters: 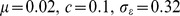.(PDF)

Figure S2The evolution of the multivariate maternal effects matrix 
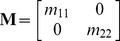
 when fluctuations in 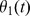 and 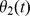 are periodic (panel A) or stochastic (panel B). Panel A: Similar to the univariate scenario depicted in Figure 1, we find that *m*_11_ and *m*_22_ evolve to positive values when periodic fluctuations are slow, whereas maternal effects evolve to be negative when fluctuations occur rapidly. Panel B: when the autocorrelation in selective conditions is positive (negative) between two subsequent timesteps, *m*_11_ and *m*_22_ again evolve to positive (negative) values, although magnitudes are more modest relative to panel A. Note that both selective optima are identical 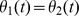 in the periodic environment, whereas both optima are uncorrelated in the stochastic environment. Parameters: 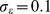, 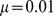, 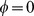, 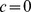 (panel A), 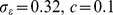 (panel B).(PDF)

Figure S3The evolution of multivariate maternal effects in a stochastically fluctuating environment, where 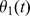 and 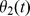 are Gaussian random variables. Panels A–C: when both optima are identical (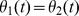) we find alternative stable states, similar to the ones found in the periodic environment in Figure 3. Panel D–E: both optima are uncorrelated, yet identically distributed. Consequently, the alternative stable states collapse to a single outcome. Parameters: 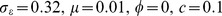.(PDF)

Figure S4An example simulation showing the selective advantage of evolving maternal effects in a periodically fluctuating environment, when the rate of environmental change is 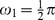 (see also Figures 3A,B). At first, only the genetic values **a**(*t*) that code for phenotypes *z*_1_ and *z*_2_ are allowed to evolve, to obtain a baseline measure of adaptation to a fluctuating environment in terms of the number of surviving individuals 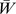 (panel C). From generation 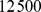 onwards, the two same-trait maternal effects *m*_11_ and *m*_22_ are allowed to evolve in addition to both genetic values. However, panels B and C show that *m*_11_ and *m*_22_ do not enhance adaptation to a fluctuating environment. This is unsurprising, as the two same-trait maternal effects *m*_11_ and *m*_22_ can only lead to fluctuations in which phenotypes change sign at every generation (i.e., when 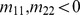). However, for the rate of environmental change 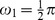 considered here, the optimal phenotype would need to change sign every second generation (see main text). When also both cross-trait maternal effects *m*_12_ and *m*_21_ are allowed to evolve (from generation 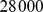 onwards), increased flexibility allows for phenotypic adaptation to the fluctuating environment (panel B), thereby eliminating the deep troughs of the fitness landscape in panel C. Parameters: 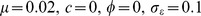.(PDF)

Figure S5The evolution of multivariate maternal effects in a stochastically fluctuating environment, where the selective optimum 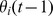 in the parental generation is cross-correlated with 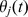 in the offspring generation. Panel A: the cross-correlation between optimum 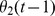 and 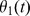 is varied from −1 to 1, while the other cross-correlation 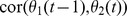 is constrained between values 0 and −0.1. As a consequence, the cross-trait maternal effect *m*_12_ from maternal trait 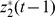 to offspring trait 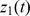 evolves from negative to positive values, in line with the dominant cross-correlation. Panel B: now, the cross-correlation between optimum 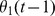 and 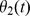 is varied from −1 to 1, while the other cross-correlation is constrained to much smaller values. Again, we find that the corresponding cross-trait maternal effect *m*_21_ from maternal trait 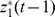 to offspring trait 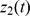 evolves from negative to positive, in line with the cross-correlation. In both panels, the within-trait autocorrelations 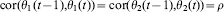 are set at *ρ* = 0.1. Parameters: 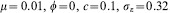.(PDF)
