# Supplementary material for: The Evolution of Multivariate Maternal Effects
Source: PLoS Comput Biol. 2014 Apr 10;10(4):e1003550. doi: 10.1371/journal.pcbi.1003550 (PMC3983079; doi:10.1371/journal.pcbi.1003550)
Supplement: Figure S1 — The evolution of a single maternal effect m when fluctuations in θ(t) are stochastic rather than periodic. Instead of varying the frequency of environmental change ω 1 (which is only relevant to periodic environments), we now vary the autocorrelation ρ between selective conditions experienced by mother and offspring. Maternal effects evolve to be positive when ρ attains large, positive values. By contrast, negative values evolve for smaller values of ρ. Each dot represents the average maternal effect measured over ten replicate simulations (at generation ), while the shaded areas depict corresponding standard deviations. Parameters: . (PDF) [file pcbi.1003550.s001.pdf]

Figure S1 The evolution of a single maternal effect m when fluctuations in $\theta(t)$ are stochastic rather than periodic. Instead of varying the frequency of environmental change ω_1 (which is only relevant to periodic environments), we now vary the autocorrelation ρ between selective conditions experienced by mother and offspring. Maternal effects evolve to be positive when ρ attains large, positive values. By contrast, negative values evolve for smaller values of ρ. Each dot represents the average maternal effect$\bar{m}$ measured over ten replicate simulations (at generation $t = 50000$), while the shaded areas depict corresponding standard deviations. Parameters: $\mu = 0.02, c = 0.1, \sigma_\varepsilon = 0.32$.

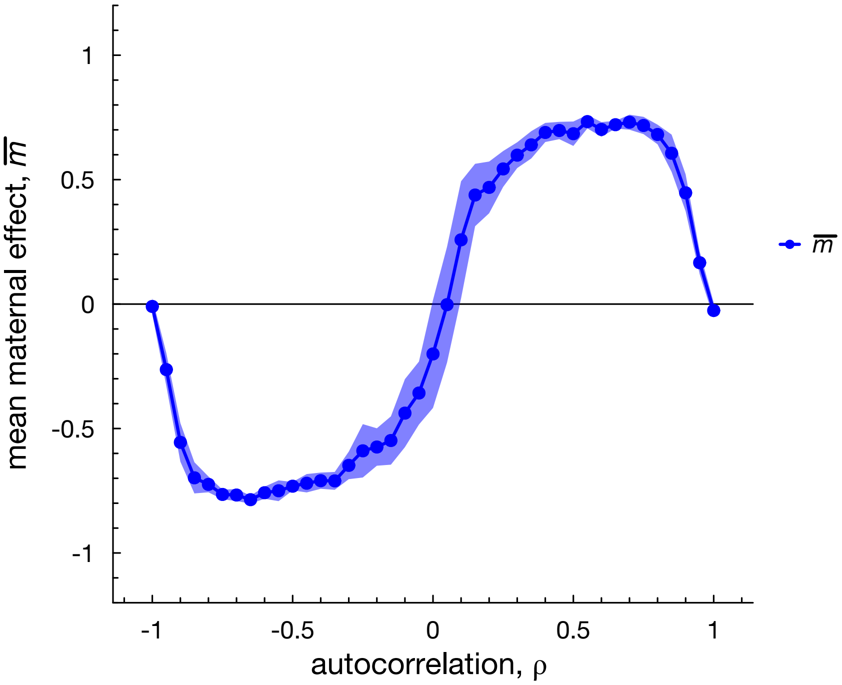

Figure S1:
