# Supplementary material for: The Evolution of Multivariate Maternal Effects
Source: PLoS Comput Biol. 2014 Apr 10;10(4):e1003550. doi: 10.1371/journal.pcbi.1003550 (PMC3983079; doi:10.1371/journal.pcbi.1003550)
Supplement: Figure S2 — The evolution of the multivariate maternal effects matrix when fluctuations in and are periodic (panel A) or stochastic (panel B). Panel A: Similar to the univariate scenario depicted in Figure 1, we find that m 11 and m 22 evolve to positive values when periodic fluctuations are slow, whereas maternal effects evolve to be negative when fluctuations occur rapidly. Panel B: when the autocorrelation in selective conditions is positive (negative) between two subsequent timesteps, m 11 and m 22 again evolve to positive (negative) values, although magnitudes are more modest relative to panel A. Note that both selective optima are identical in the periodic environment, whereas both optima are uncorrelated in the stochastic environment. Parameters: , , , (panel A), (panel B). (PDF) [file pcbi.1003550.s002.pdf]

Figure S2 The evolution of the multivariate maternal effects matrix $\mathbf{M} = \begin{bmatrix} m_{11} & 0 \\ 0 & m_{22} \end{bmatrix}$ when fluctuations in $\theta_1(t), \theta_2(t)$ are periodic (panel A) or stochastic (panel B). Panel A: Similar to the univariate scenario depicted in Figure 2, we find that m_{11} and m_{22} evolve to positive values when periodic fluctuations are slow, whereas maternal effects evolve to be negative when fluctuations occur rapidly. Panel B: when the autocorrelation in selective conditions is positive (negative) between two subsequent timesteps, m_{11} and m_{22} again evolve to positive (negative) values, although magnitudes are more modest relative to panel A. Note that both selective optima are identical $\theta_1(t) = \theta_2(t)$ in the periodic environment, whereas both optima are uncorrelated in the stochastic environment. Parameters: $\mu = 0.01$, $\phi = 0$ and $\sigma_\varepsilon = 0.1$, $c = 0$ (panel A), $\sigma_\varepsilon = 0.32$, $c = 0.1$ (panel B).

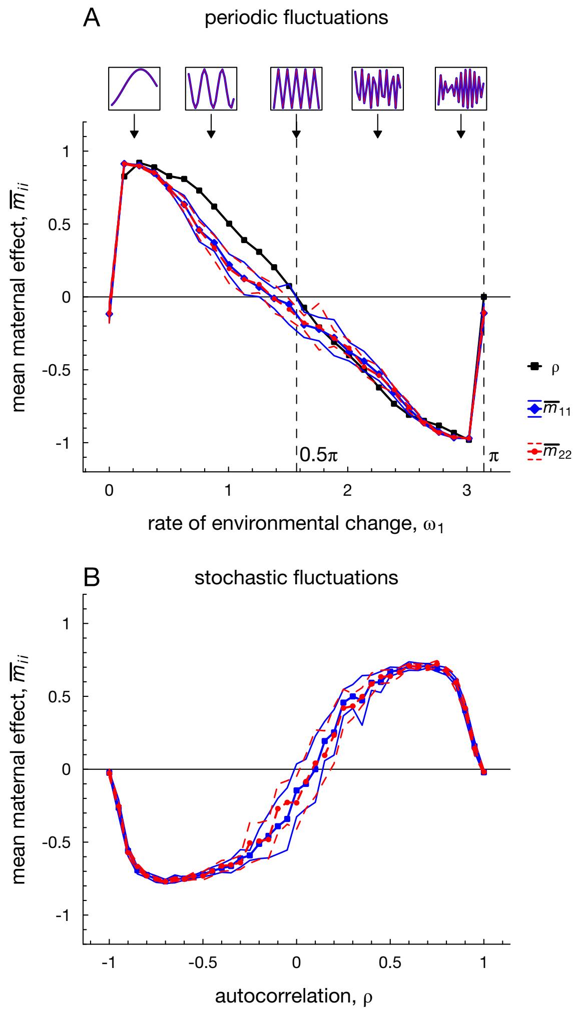

Figure S2:
