# Supplementary material for: The Evolution of Multivariate Maternal Effects
Source: PLoS Comput Biol. 2014 Apr 10;10(4):e1003550. doi: 10.1371/journal.pcbi.1003550 (PMC3983079; doi:10.1371/journal.pcbi.1003550)
Supplement: Figure S3 — The evolution of multivariate maternal effects in a stochastically fluctuating environment, where and are Gaussian random variables. Panels A–C: when both optima are identical () we find alternative stable states, similar to the ones found in the periodic environment in Figure 3. Panel D–E: both optima are uncorrelated, yet identically distributed. Consequently, the alternative stable states collapse to a single outcome. Parameters: . (PDF) [file pcbi.1003550.s003.pdf]

Figure S3 The evolution of multivariate maternal effects in a stochastically fluctuating environment, where $\theta_1(t)$ and $\theta_2(t)$ are Gaussian random variables. Panels A-C: when both optima are identical ($\theta_1(t) = \theta_2(t)$) we again find alternative stable states as in Figure 3 in the main text. Panel D-E: both optima are uncorrelated, yet identically distributed. Consequently, the alternative stable states collapse to a single outcome. Parameters: $\sigma_\varepsilon = 0.32$, $\mu = 0.01$, $\phi = 0$, $c = 0.1$.

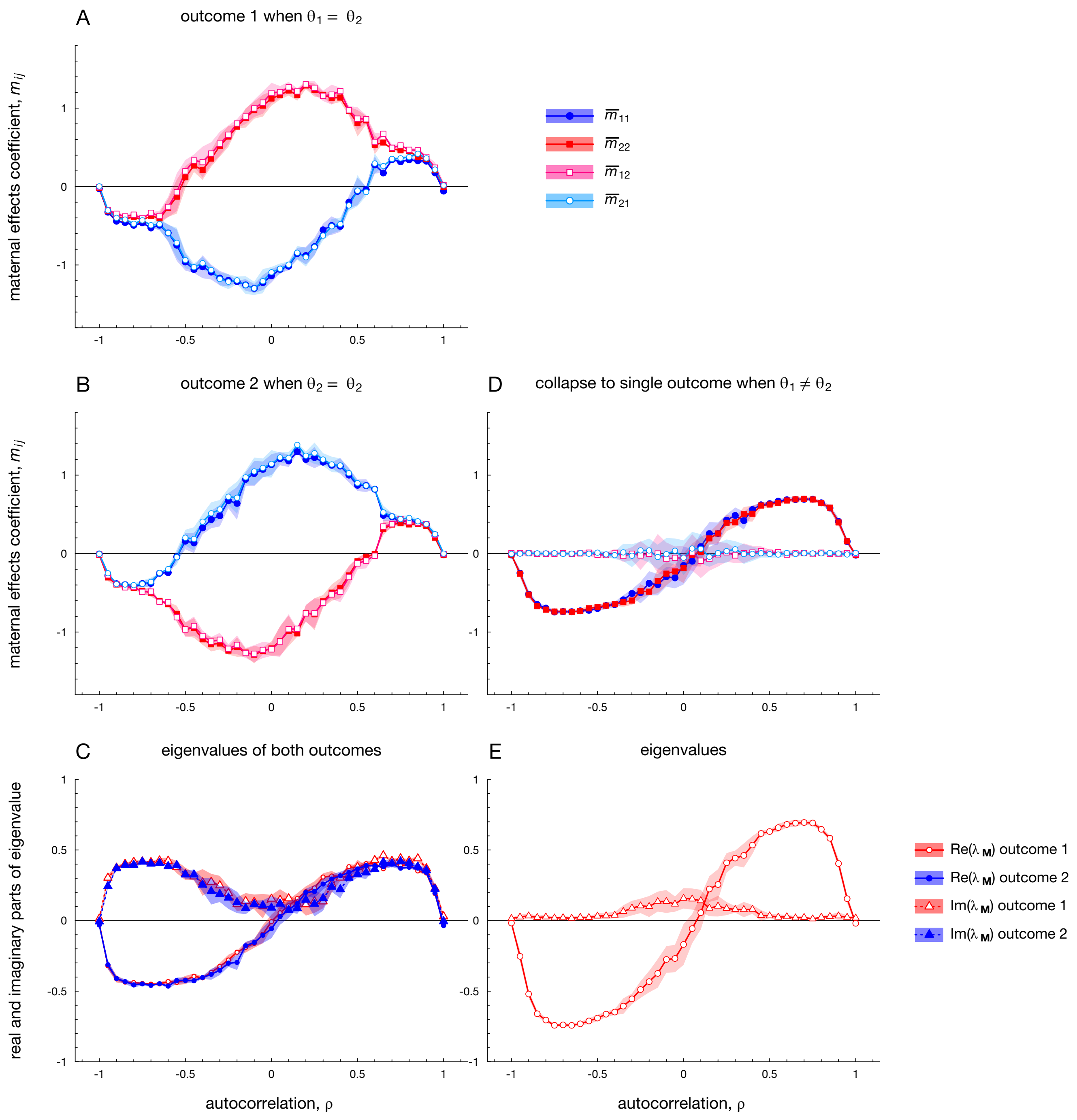

A
outcome 1 when θ1 = θ2
maternal effects coefficient, m_{ij}
$\overline{m}_{11}$
$\overline{m}_{22}$
$\overline{m}_{12}$
$\overline{m}_{21}$
B
outcome 2 when θ2 = θ2
D
collapse to single outcome when θ1 ≠ θ2
C
eigenvalues of both outcomes
real and imaginary parts of eigenvalue
E
eigenvalues
Re(λ M) outcome 1
Re(λ M) outcome 2
Im(λ M) outcome 1
Im(λ M) outcome 2
autocorrelation, ρ

Figure S3:
