# Supplementary material for: The Evolution of Multivariate Maternal Effects
Source: PLoS Comput Biol. 2014 Apr 10;10(4):e1003550. doi: 10.1371/journal.pcbi.1003550 (PMC3983079; doi:10.1371/journal.pcbi.1003550)
Supplement: Figure S4 — An example simulation showing the selective advantage of evolving maternal effects in a periodically fluctuating environment, when the rate of environmental change is (see also Figures 3A,B). At first, only the genetic values a(t) that code for phenotypes z 1 and z 2 are allowed to evolve, to obtain a baseline measure of adaptation to a fluctuating environment in terms of the number of surviving individuals (panel C). From generation onwards, the two same-trait maternal effects m 11 and m 22 are allowed to evolve in addition to both genetic values. However, panels B and C show that m 11 and m 22 do not enhance adaptation to a fluctuating environment. This is unsurprising, as the two same-trait maternal effects m 11 and m 22 can only lead to fluctuations in which phenotypes change sign at every generation (i.e., when ). However, for the rate of environmental change considered here, the optimal phenotype would need to change sign every second generation (see main text). When also both cross-trait maternal effects m 12 and m 21 are allowed to evolve (from generation onwards), increased flexibility allows for phenotypic adaptation to the fluctuating environment (panel B), thereby eliminating the deep troughs of the fitness landscape in panel C. Parameters: . (PDF) [file pcbi.1003550.s004.pdf]

Figure S4 An example simulation showing the selective advantage of evolving maternal effects in a periodically fluctuating environment, when the rate of environmental change is $\omega_1 = \frac{1}{2}\pi$ (see also Figures 3A,B). At first, only the genetic values $\mathbf{a}(t)$ that code for phenotypes z_1 and z_2 are allowed to evolve, to obtain a baseline measure of adaptation to a fluctuating environment in terms of the number of surviving individuals $\bar{W}$ (panel C). From generation 12 500 onwards, the two same-trait maternal effects m_{11} and m_{22} are allowed to evolve in addition to both genetic values. However, panels B and C show that m_{11} and m_{22} do not enhance adaptation to a fluctuating environment. This is unsurprising, as the two same-trait maternal effects m_{11} and m_{22} can only lead to fluctuations in which phenotypes change sign at every generation (i.e., when $m_{11}, m_{22} < 0$). However, for the rate of environmental change $\omega_1 = \frac{1}{2}\pi$ considered here, the optimal phenotype would need to change sign every second generation (see main text). When also both cross-trait maternal effects m_{12} and m_{21} are allowed to evolve (from generation 28 000 onwards), increased flexibility allows for phenotypic adaptation to the fluctuating environment (panel B), thereby eliminating the deep troughs of the fitness landscape in panel C. Parameters: $\mu = 0.02$, $c = 0$, $\phi = 0$, $\sigma_\varepsilon = 0.1$.

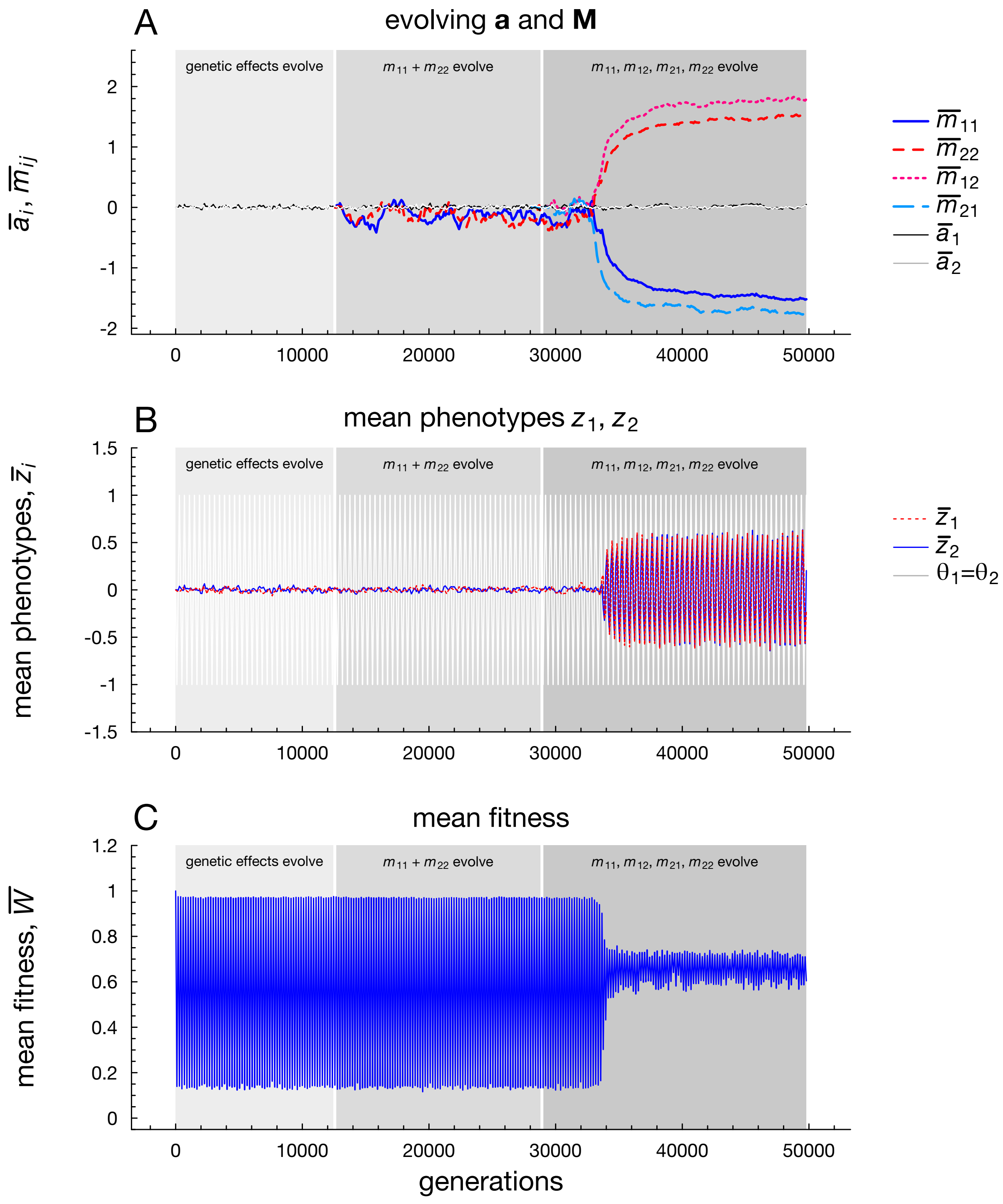
A
evolving **a** and **M**
genetic effects evolve
m_{11} + m_{22} evolve
m_{11}, m_{12}, m_{21}, m_{22} evolve
$\bar{a}_i$, $\bar{m}_{ij}$
$\bar{m}_{11}$
$\bar{m}_{22}$
$\bar{m}_{12}$
$\bar{m}_{21}$
$\bar{a}_1$
$\bar{a}_2$
B
mean phenotypes z_1, z_2
mean phenotypes, $\bar{z}_i$
$\bar{z}_1$
$\bar{z}_2$
θ_1=θ_2
C
mean fitness
mean fitness, $\overline{W}$
generations

Figure S4:
