# Supplementary material for: The Evolution of Multivariate Maternal Effects
Source: PLoS Comput Biol. 2014 Apr 10;10(4):e1003550. doi: 10.1371/journal.pcbi.1003550 (PMC3983079; doi:10.1371/journal.pcbi.1003550)
Supplement: Figure S5 — The evolution of multivariate maternal effects in a stochastically fluctuating environment, where the selective optimum in the parental generation is cross-correlated with in the offspring generation. Panel A: the cross-correlation between optimum and is varied from −1 to 1, while the other cross-correlation is constrained between values 0 and −0.1. As a consequence, the cross-trait maternal effect m 12 from maternal trait to offspring trait evolves from negative to positive values, in line with the dominant cross-correlation. Panel B: now, the cross-correlation between optimum and is varied from −1 to 1, while the other cross-correlation is constrained to much smaller values. Again, we find that the corresponding cross-trait maternal effect m 21 from maternal trait to offspring trait evolves from negative to positive, in line with the cross-correlation. In both panels, the within-trait autocorrelations are set at ρ = 0.1. Parameters: . (PDF) [file pcbi.1003550.s005.pdf]

Figure S5 The evolution of multivariate maternal effects in a stochastically fluctuating environment, where the selective optimum $\theta_i(t-1)$ in the parental generation is correlated with $\theta_j(t)$ in the offspring generation. Panel A: the cross-correlation between optimum $\theta_2(t-1)$ and $\theta_1(t)$ is varied from -1 to 1, while the other cross-correlation $\text{cor}(\theta_1(t-1), \theta_2(t))$ is constrained between values 0 and –0.1. As a consequence, the cross-trait maternal effect m_{12} from maternal trait $z_2^*(t-1)$ to offspring trait $z_1(t)$ evolves from negative to positive values, in line with the dominant cross-correlation. Panel B: now, the cross-correlation between optimum $\theta_1(t-1)$ and $\theta_2(t)$ is varied from -1 to 1, while the other cross-correlation is constrained to much smaller values. Again, we find that the corresponding cross-trait maternal effect m_{21} from maternal trait $z_1^*(t-1)$ to offspring trait $z_2(t)$ evolves from negative to positive, in line with the cross-correlation. In both panels, the within-trait autocorrelations $\text{cor}(\theta_1(t-1), \theta_1(t)) = \text{cor}(\theta_2(t-1), \theta_2(t)) = \rho$ are set at $\rho = 0.1$. Parameters: $\mu = 0.01$, $\phi = 0$, $c = 0.1$, $\sigma_\varepsilon = 0.32$.

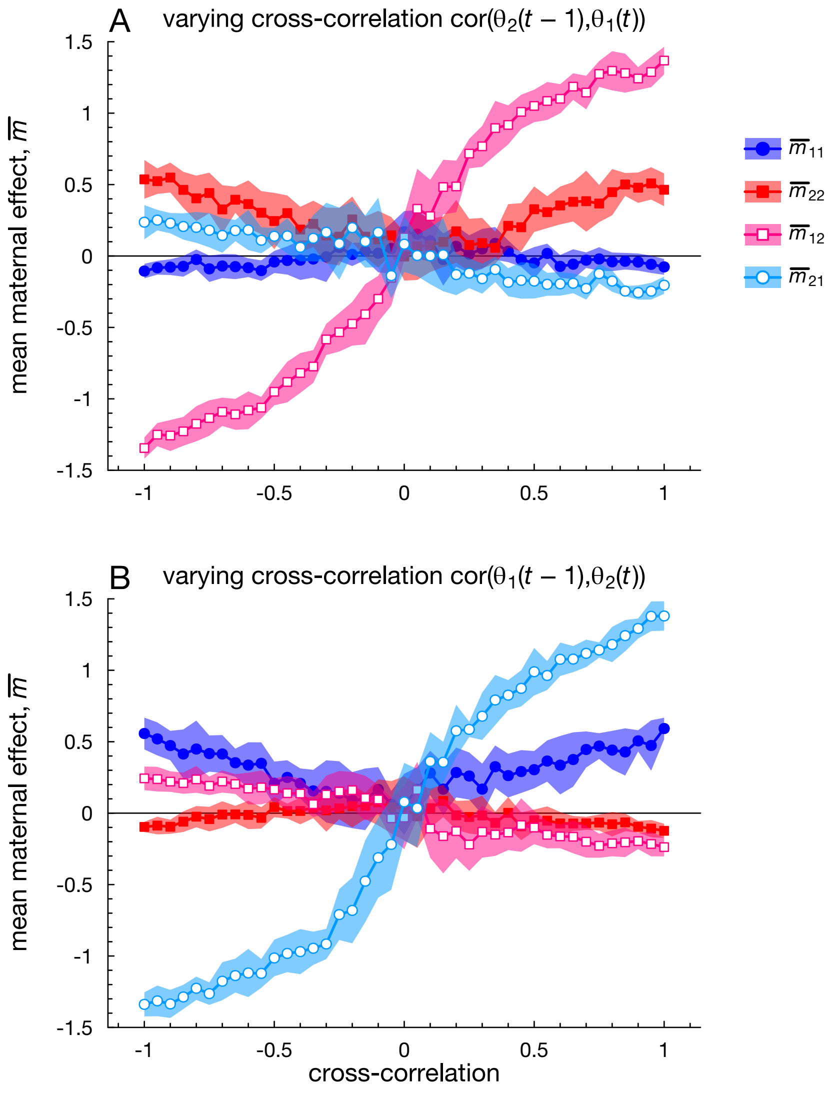
A
varying cross-correlation cor(θ2(t − 1),θ1(t))
mean maternal effect, m̄
m̄11
m̄22
m̄12
m̄21
B
varying cross-correlation cor(θ1(t − 1),θ2(t))
cross-correlation

Figure S5:
